# Towards super‐resolved terahertz microscopy for cellular imaging

**DOI:** 10.1111/jmi.13132

**Published:** 2022-07-19

**Authors:** Rocco D'Antuono, John W. Bowen

**Affiliations:** ^1^ Crick Advanced Light Microscopy STP The Francis Crick Institute 1 Midland Road, NW1 1AT London UK; ^2^ Department of Biomedical Engineering, School of Biological Sciences University of Reading Reading UK

**Keywords:** cellular, deconvolution, imaging, microscopy, super‐resolution, terahertz

## Abstract

Biomedical imaging includes the use of a variety of techniques to study organs and tissues. Some of the possible imaging modalities are more spread at clinical level (CT, MRI, PET), while others, such as light and electron microscopy are preferred in life sciences research. The choice of the imaging modalities can be based on the capability to study functional aspects of an organism, the delivered radiation dose to the patient, and the achievable resolution.

In the last few decades, spectroscopists and imaging scientists have been interested in the use of terahertz (THz) frequencies (30 μm to 3 mm wavelength) due to the low photon energy associated (*E*∼1 meV, not causing breaking of the molecular bonds but still interacting with some vibrational modes) and the high penetration depth that is achievable. THz has been already adopted in security, quality control and material sciences. However, the adoption of THz frequencies for biological and clinical imaging means to face, as a major limitation, the very scarce resolution associated with the use of such long wavelengths. To address this aspect and reconcile the benefit of minimal harmfulness for bioimaging with the achievable resolving power, many attempts have been made. This review summarises the state‐of‐the‐art of THz imaging applications aimed at achieving super‐resolution, describing how practical aspects of optics and quasi‐optics may be treated to efficaciously implement the use of THz as a new low‐dose and versatile modality in biomedical imaging and clinical research.

## 1 INTRODUCTION

### 1.1 Imaging modalities for biological and clinical samples: practical technical aspects

Biomedical imaging is the application of sensing and imaging techniques to produce image‐based representations of biological and clinical samples. The life sciences and medicine strongly rely on a variety of imaging modalities such as electron microscopy (EM), light microscopy (LM), magnetic resonance imaging (MRI), positron emission tomography (PET) and computed tomography (CT). Among the aforementioned modalities adopted in clinical practice, some are characterised by a low radiation dose delivered to the imaged tissues (LM, MRI),^1^ while others imply either the use of radioactive tracers (PET)^2^ or need an accurate instrument calibration (CT) to minimise the potential risks of patient exposure.^3^, ^4^ Furthermore, the running cost, the distribution on the local territory and the requirement of qualified operators may constitute a limiting factor for the application of some medical imaging devices.^5^

An important aspect to consider in selecting the appropriate imaging technique is the achievable resolution, defined as the size of the minimum detail that is visible through the use of a particular technique. The need for a specific level of detail can be context‐specific. For instance, before clinical surgery, it might be satisfactory to determine the size of a neoplastic formation with an imaging modality that achieves sub‐millimetre resolution, such as the CT. In the context of life sciences, instead, the objects of investigation are often single cells and organelles. Then, the techniques commonly adopted are LM and EM, which respectively allow sub‐micron^6^ and sub‐nanometre resolution.^7^ The achievable resolution may also be dependent on the acceptable patient dose, as in the choice of the ‘pitch’ for CT^8^ and the strength of the magnetic field gradient for MRI,^9^ or it can depend on the wavelength of electromagnetic beam used, as in LM. Therefore, the imaging of a biological sample implies an accurate balance between the desired level of detail (resolution) and sample preservation (patient body, ex vivo tissue, cellular/subcellular structure etc.).

A second aspect to be considered for potential clinical applications is the imaging penetration depth. For instance, if light (electromagnetic radiation) is used to investigate fluorescence, scattering or transmitted intensity, the sample thickness constitutes the main limitation in obtaining the correct information.

Some of the required conditions for imaging a tissue are a compromise between a sufficient signal‐to‐background ratio, meaning that the structures of interest are well highlighted compared to the rest of the tissue, and a reasonable acquisition time, to minimise the patient annoyance. For example, in CT, the attenuation of the X‐ray beam passing through tissues is measured using as reference the attenuation caused by the passage through the equivalent length of water and the contrast between bone and muscle is determined by the different attenuation ranges, 800–3000 for the first and 35–50 for the latter.^8^

Regarding the acquisition time, several clinical imaging techniques imply the necessity of sessions lasting from a few minutes to more than an hour,^10^ during which the patient may be required to follow specific ‘breath‐hold’ procedures^11^ or fitted with constraining equipment to minimise motion artefacts.^12^

The choice of a specific imaging modality depends also on the target tissue, especially in relation to the achievable imaging depth. For instance, although MRI may require more expensive equipment compared to an episcopic application of LM,^13^ the first is able to supply insight of the organs (such us brain or abdominal cavity), while the second is affected by the high extinction in tissues. Indeed, the penetration of visible light through biological samples is wavelength‐dependent and limited to a few millimetres. In particular, a beam containing visible light wavelengths that passes through human skin decreases, on average, to around 50% of the incident intensity after just 80 μm.^14^ On the other hand, the use of X‐rays in CT allows the penetration through both soft and hard biological tissues but, as already mentioned, it has to be limited to indispensable cases due to the high radiation dose delivered to patients. It has been estimated that CT scans may account for up to 90% of the dose from artificial sources to which humans are exposed.^4^

### 1.2 Correlative methods for biological and preclinical imaging

Both in clinical and biological imaging, there has been an important advancement in the adoption of multiple techniques to image the same sample for the purpose of studying different functional features of a tissue, cell type or molecular structure. This approach is classified as correlative imaging.^15^ In this regard, it is common practice in medical imaging to couple PET, CT and MRI in different combinations,^16^ while in bioimaging, LM and EM sample preparations have been made compatible with a method known as correlative light‐electron microscopy (CLEM).

In this instance, the LM allows functional information derived by fluorescence staining to be combined with the ultrastructure resolution of the EM. The correlation accuracy between the two techniques has been improved down to around 10–20 nm.^17^ Some of the aforementioned correlative methods are only applicable on fixed samples (CLEM), whereas others might include a non‐irrelevant radiation dose for the patient. Therefore, for medical and live biological applications, it is fundamental to identify correlative methods able to combine multiple desirable characteristics such as low dose, sufficient tissue penetration, good optical resolution and decent tissue‐induced contrast.

### 1.3 Considerations for the adoption of terahertz (THz) frequencies in biological and clinical imaging

**FIGURE 1:**
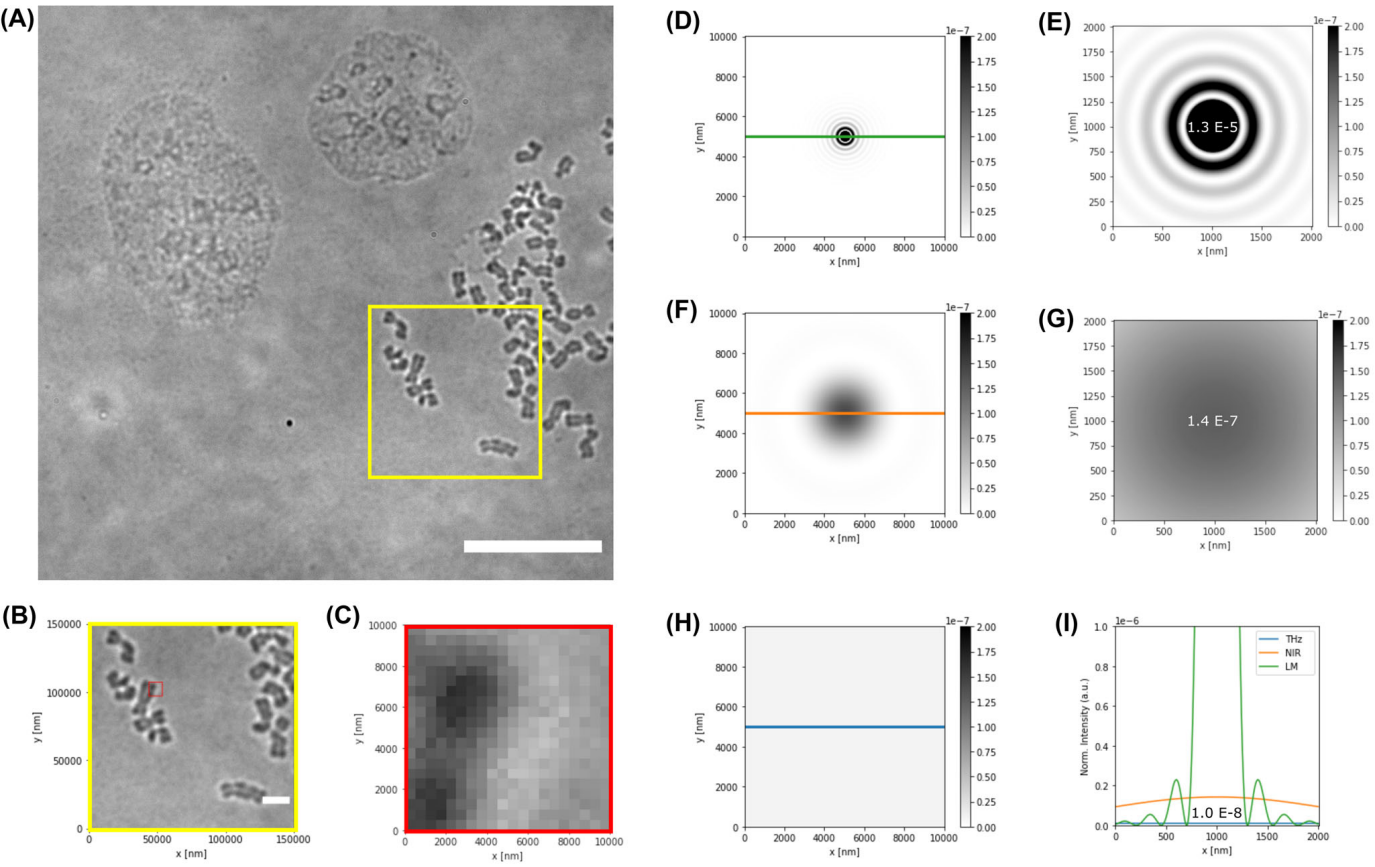
Optical resolution and Airy patterns for LM, NIR and THz radiation compared with typical cell size. The imaging of biological structures such as cell nuclei or organelles, with techniques such as LM, generates diffraction‐limited images with intensity resulting from the superposition of Airy patterns generated by the individual point‐like sources in the real object. According to the Rayleigh criterion two identical objects are said to be resolved, in an image, if their corresponding diffraction patterns are at a distance greater than their Airy disk radius. Biological structures acquired with LM modality are compared with simulated diffraction patterns obtainable using different radiation wavelengths. Airy patterns were generated with Astropy package.^59^ (A) LM image of HB2 cell nuclei and condensed chromosomes (yellow square), acquired in transmitted mode. Scale bar: 120 μm. (B) Condensed chromosomes and subregion of a single chromatid (red square). Scale bar: 20 μm. (C) LM image detail shown at single‐pixel level (pixel size: 0.52 μm). (D) Simulated diffraction pattern with Airy disk radius of 300 nm, comparable to that obtained with state‐of‐the‐art lenses in LM. (E) Detail of (D) showing the normalised maximum intensity peak *I*_Max, LM_ = 1.3 × 10^−5^ (normalisation: integral of Airy pattern intensity set equal to 1). (F) Simulated diffraction pattern with Airy disk radius of 3000 nm, if wavelength in the NIR were used for imaging. (G) Detail of (F) showing how the normalised maximum intensity peak *I*_Max_, _NIR_ = 1.4 × 10^−7^ is two orders of magnitude lower than the one corresponding to LM. (H) Simulated diffraction pattern with Airy disk radius of 118,800 nm (118.8 μm), corresponding to typical imaging conditions for THz imaging. (I) Normalised intensity profiles for the LM, NIR and THz wavelengths considered in D–H. Over the considered field of view where chromosome details are visible (image C, size:10 μm × 10 μm), the intensity pattern of THz diffracted radiation shows a broadened peak (*I*_Max, THz_ = 1.0 × 10^−8^) that does not allow features to be distinguished according to the Rayleigh criterion for optical resolution. To use THz for biological imaging at cellular scales it is therefore necessary to adopt specific super‐resolution methods for quasi‐optical (i.e. THz) beams

The inclusion of terahertz (THz) imaging in biology and clinical practice seems to open new horizons for correlative methods. Indeed, compared to LM in the visible range, THz radiation is characterised by longer wavelengths and a wider frequency range, spanning between 100 GHz and 10 THz (corresponding to the range of wavelengths between λ = 3 mm and λ = 30 μm).^18^ The large interval of wavelengths and frequencies may be advantageous since it allows discrimination of molecular species in a sample on the basis of extinction coefficient. However, when compared to LM in the visible range, the resolving power of THz radiation is hugely limited by the corresponding longer wavelengths. In fact, the optical resolution is defined by Abbe's law, which sets the minimum object size that is detectable to be directly proportional to the wavelength of the used radiation.^6^ Unfortunately, if the goal is to study cellular and subcellular compartments, the typical sizes considered are in the order of a few micrometres or below (Figure 1A–C). The Rayleigh criterion establishes that two equal point‐like objects are said to be resolved if their distance in the image is greater than the radius of the Airy disk in the diffraction pattern that they generate.^6^ The profile of the diffraction pattern and the size of the central Airy disk depends on the lens used through parameters such its diameter, its focal distance and the light wavelength. For a good lens the size of the diffraction pattern can be considered to range between *λ*/2 and 2*λ*.^19^ Therefore, the resolution limit, in principle, increases linearly with the wavelength of the used electromagnetic radiation (Figure 1D–H). The size of the Airy disk generated focusing a quasi‐optical beam in the THz frequencies, then, hugely offsets the resolution that is achievable (Figure 1I), limiting it to hundreds of micrometres.^20^ Thus, the development of THz applications for biological imaging implies the need to address the scarce resolution issue.

## 2 INFRARED RADIATION IMAGING AND SPECTROSCOPY

### 2.1 Infrared imaging

The infrared (IR) region of the electromagnetic spectrum can be classified accordingly to the International Standard ISO 20473 as near infrared (NIR) λ = 0.78–3 μm, mid INFRARED (MIR) λ = 3–50 μm and far infrared (FIR) λ = 50–1000 μm.^21^

In general, the use of longer wavelengths (compared to the visible range) of the electromagnetic spectrum for imaging and potential therapeutic applications allows achievement of a higher penetration depth or matching to the energy levels associated with the activity of biological molecules (Table 1).

**TABLE 1:** Wavelength range, photon energy, resolution, penetration depth, biological interaction mechanisms and observed phototoxicity of LM, NIR and THz

	Wavelength range (μm)	Photon energy (eV)	Achieved resolution (μm)*	Penetration depth (μm)**	Interaction mechanisms	Phototoxicity	References
LM	0.4–0.7	3.1–1.77	0.2–1.4	400–2500	Electronic transitions Conformational changes	Reduced progress into mitosis Cell death	^60^, ^61^, ^62^
NIR	0.78–3	1.59–0.41	0.39–6	420–4230	Electronic transitions Heat Stretching of O‐H, CH_3_, CH_2_	ROS production Temperature and HSP increase Autophagy	^63^ ^,^ ^64^ ^,^ ^27^
THz	30–3000	4.1 × 10^−2^ to 4.1 × 10^−4^	15–6000	Skin: ∼400–1100 Fat: ∼3500–9800	H_2_O vibrational modes Hydrogen bond O‐H polarity	DNA demethylation Reduced actin polymerisation DNA base pairing dynamics Apoptosis and necrosis	^65^ ^,^ ^46^

* Considering the size of the diffraction pattern first disk ranging between *λ*/2 and 2*λ* (see Section 1.3).

** Depth in biological tissue at which intensity decreases to 1/*e* of the incident beam. Range determined considering minimum and maximum for different wavelengths and types of murine and human ex vivo tissues.

*Note*: Comparison between commonly used wavelength ranges (LM and NIR) with THz, for microscopy applications. Electromagnetic radiation of different wavelengths generates diffraction spots with variable size when focused by lenses; according to the Rayleigh criterion for optical resolution, this sets the limit for the achievable resolution. The radiation wavelength also influences the penetration depth through biological tissues due to scattering and interaction mechanisms with biological molecules, which are determined by the associated photon energy. Literature reports the presence of phototoxic effects caused by the use of both LM and NIR. Some studies regarding THz effects on cells and biological molecules have determined possible undesirable effects such as apoptosis and necrosis, beside interesting applications that include the control of actin polymerisation, DNA methylation and base‐pair dynamics.

NIR imaging is currently considered a well‐integrated option in intravital microscopy, since it can be easily deployed on point‐scanning laser systems, in conjunction with visible range laser lines that are adopted for confocal microscopy. The advantage of using NIR tuneable pulsed lasers relies on the possibility of visualising in‐depth fluorescent tissues^22^ and to obtain, as a label‐free signal, the second harmonic generation (SHG) from biologically relevant non‐centrosymmetric structures such as collagen^23^ and myosin.^24^ Furthermore, in multiphoton microscopy, the excitation probability decreases very rapidly as function of the distance from the focal plane, conferring then an intrinsic spatial confinement to the generated fluorescence or label‐free (SHG) signal; indeed, the effective excitation volume is in the order of a femtolitre.^25^

### 2.2 Infrared spectroscopic applications

Other applications of IR radiation include a variety of spectroscopic techniques to study molecular vibrational and rotational modes.^26^

In this regard, Fourier Transform infrared (FTIR) spectroscopy allows the identification of molecules and chemical bonds by the spectrum of absorbed wavelengths. The typical wavenumber range for the investigation of biological molecules is 1800−900 cm^−1^,^27^ corresponding approximately to the wavelength range of 5–11 μm. FTIR has been used for very different purposes including the identification and classification of bacteria^28^ or the determination of bone composition properties such as the mineral content or the collagen maturity.^29^ Furthermore, it can be employed as a microscopy technique, but the sample thickness cannot exceed a few micrometres and the hydration has to be low enough to avoid the relevant IR absorption caused by the water molecules.^27^ On the other hand, Raman spectroscopy, based on the measurement of inelastic scattering of NIR radiation, is applicable to abundantly hydrated samples such as tissue slices but is characterised by a lower signal‐to noise compared to FTIR and subject to the spectral distortion or the high background originated by the relatively strong tissue autofluorescence.^29^

Then, it seems that water, the quintessential molecule type composing biological tissues, might represent a source of trouble for some IR imaging and spectroscopy techniques.

The presence in the human body of highly sensitive mechanisms for light and heat detections, such as the measurable hyperpolarisation of a rod cell upon the absorption of a single photon or the response of skin sensory cells to an increase in temperature as small as 0.01 K,^19^, ^30^ has inspired the investigation of other types of external electromagnetic fields able to interact with biological processes.

Beyond the IR, lower radio frequencies (RF) are widely used in telecommunications. Regrettably, numerous pieces of evidence have excluded the possibility that RF with frequencies above ν ≥ 10 MHz can interact with biological systems. Moreover, the energy associated with RF fields (*E* = 4.0 × 10^−4^ kJ/mol for a photon of frequency *ν* = 1 GHz) and the possible local focussing effect of tissue layers acting like dielectric lenses seem not to be able to generate any microthermal effect, that is, increase the local temperature more than the thermal background in a body at 310 K (*E* = 2.6 kJ/mol).^19^

Possible interactions between fields and biological molecules should then be identified at higher frequencies in the IR and perhaps through mechanisms involving water molecules.^19^ Indeed, water (H_2_O) inside and around proteins contributes to stabilise the structure with hydrogen bonds and modulate the chemical reactions through the topology of the hydration layers (shells).^31^ Measurements of dielectric dispersion have been used to quantify the extent of those water shells for relevant biomolecules; for example, the dielectric behaviour of the commonly used BSA protein in water has been studied in the frequency range MHz to THz. By using the superposition of multiple Debye relaxations, it has been possible to quantify the fractions of bulk, loosely bound and tightly bound water components, finding relaxation times of, respectively, τ_D_ ≈ 8.27 ± 0.35 ps, τ_2_ ≈ 38 ± 11 ps, τ_1_ ≈ 361 ± 19 ps. These correspond to peaks in the sub‐THz frequencies of *ν*_D_ ≈ 19.25 ± 0.78 GHz, *ν*_2_ ≈ 4.19 ± 0.85 GHz, *ν*_1_ ≈ 0.441 ± 0.023 GHz. By probing the same BSA in solution with THz frequencies, it is possible to determine the number of protein‐bound water molecules and the size of the first hydration shell, with the adoption of the effective‐medium approximation. This model can be employed when the radiation wavelength is much bigger than the size of the studied components in the media and has been used to fit the THz wavelength absorption coefficient of the hydrated protein in solution. Counterintuitively, the absorption of BSA in water, as for other proteins, is negative in the range *ν* = 0.050–0.600 THz, while it is positive up to *ν* = 2 THz. The measurement of the dielectric loss of BSA in solution has shown a broad peak at *ν* = 1.2 THz, indicative of the fact that the H_2_O molecules tightly bound to the protein surface (estimated to be 1150 per protein, distributed in a shell of ∼7 Å) exhibit a slower relaxation dynamics compared to the bulk water.^32^

The response of biomolecules in solution to THz radiation constitutes a relevant point in support of the adoption of this frequency range for spectroscopic and microscopic analyses in the cellular environment (Table 1).

## 3 TOWARDS THZ MICROSCOPY

### 3.1 Generation of THz radiation and existing imaging methods

The adoption of THz frequencies for imaging requires addressing a number of issues caused by low resolution at longer wavelengths, frequency‐dependent absorption of biological molecules and water and the necessity of unconventional optical components.

For a coherent source of electromagnetic radiation emitting at long wavelengths (in the THz range), the beam propagation can be assumed to be a superposition of preserved Gaussian–Hermite modes. Geometrical ray tracing is an incomplete approximation to design optical systems in this frequency range. The beam divergence is proportional to the square power of the wavelength (∝ λ ^2^) and the long‐wavelength feature of THz frequencies requires a careful design of the optical path.^33^

Furthermore, different optics are needed to manipulate the radiation beam due to the strong attenuation caused by glass used for LM and NIR imaging.^34^ Commonly adopted components for THz optics are parabolic reflectors and lenses made by optically dense materials (polyethylene, polytetrafluoroethylene, polymethylpentene, picarin, silicon etc.).^35^, ^36^ Recently, the introduction of 3D‐printed optical components has made the construction of optical systems more affordable also in the THz field.^37^

There are several methods to produce THz waves, including vacuum and solid‐state electronics (able to generate an output power *P*_max_ ∼10 mW), gas and semiconductor lasers (respectively *P*_max_ ∼10^2^ mW and *P*_max_ ∼10^3^ mW) or sources pumped by laser (*P*_max_ ∼10^2^ mW). For most of them, the underlying physics is based on the electromagnetic field generated by electron motion in vacuum or solid state materials, or by the vibrational modes of molecules. The resulting THz radiation can be subsequently classified, on the basis of its temporal profile, as pulsed or continuous wave (CW).^38^, ^39^

CW systems, such as a Gunn diode, only allow intensity measurements but require a simple optical design and are available at a low cost. In contrast, more expensive pulsed sources, such as a photoconductive antenna (PCA) or a nonlinear crystal operating optical rectification, enable measurements in the time and frequency domain, also supplying information about the depth of the sample.^40^

Terahertz time‐domain spectroscopy (THz‐TDS) is based on the analysis of the response of the sample to a THz pulse in transmitted and reflected geometry. The wave amplitude and phase are analysed and the Fourier transform of the signal allows determination of the frequency spectrum.^41^

Existing THz imaging methods used to acquire images of biological and clinical samples include raster scanning systems able to achieve typically 150 μm resolution operating at *ν* = 1 THz,^42^ line scan medical imaging probes limited to 250 μm resolution^41^ or commercial camera detectors with up to 320 × 240 pixels and pixel size of 50 μm (TZcam Terahertz Camera).^43^

**FIGURE 2:**
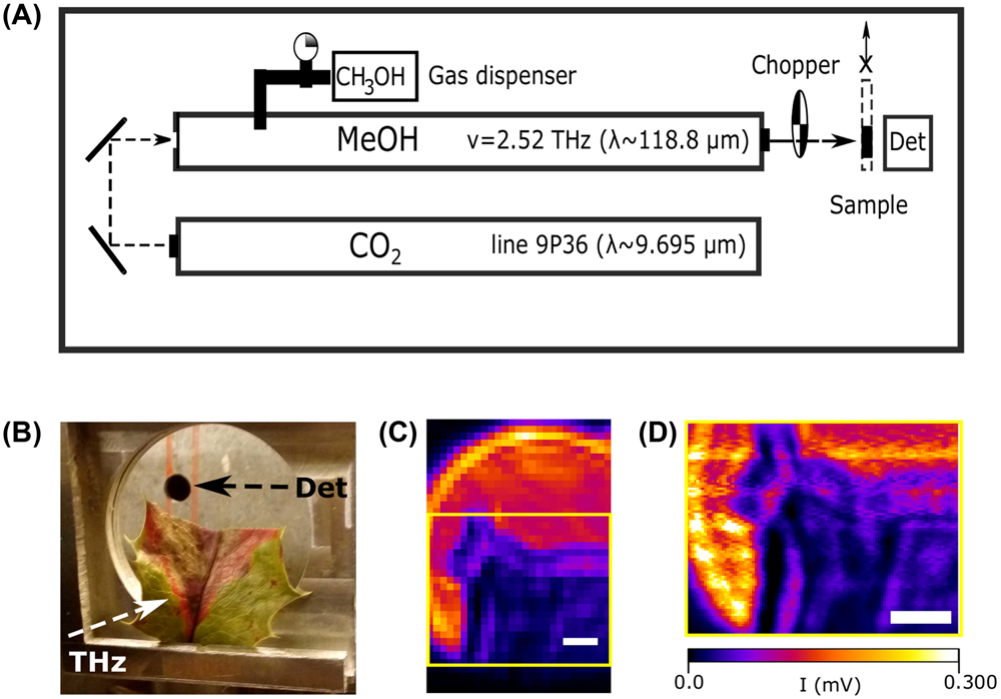
Custom raster scanning system based on an optically pumped THz laser and THz imaging of a plant leaf. (A) Scheme of the THz raster scanning system. A CO_2_ laser cavity is set to generate a pump beam at *λ* = 9.695 μm. The second cavity is filled with methanol vapour (CH_3_OH) that absorb the pump wavelength and emits THz radiation at *λ* = 118.8 μm (*ν* = 2.52 THz).^44^ The sample is scanned through the THz optically chopped beam and the signal is detected with a lock‐in amplifier. (B) Sample holder and detector are perpendicular to the incident THz beam. (C) THz image acquired in transmitted mode with pixel size corresponding to 1 mm (scale bar: 5 mm). (D) Reacquisition of (C) at higher sampling rate showing how variation in THz absorption can be measured at submillimetre scale (pixel size: 250 μm, scale bar: 5 mm)

A single‐cell imaging approach in which a near‐field PCA has been used achieved around 20 μm resolution but required more than 20 min for the acquisition of a field of view smaller than 1 mm^2^ (Li et al., 2020). Unfortunately, this level of resolving power may work for coarse‐grain tissue analysis but it is still not sufficient to allow proper THz microscopy with cellular resolution, and acquisition speed is currently far from being competitive with other imaging modalities. Figure 2A shows an example of a THz imaging system based on a custom‐made raster scanning system that uses a FIR laser pumped by a CO_2_ laser.^44^ The sample shown in Figure 2B has been acquired using THz radiation with frequency *ν* = 2.52 THz (*λ* = 118.8 μm) (Figure 2C). The image reacquired with higher sampling rate (Figure 2D) highlights how certain features of the sample, such as the exsiccated areas of the leaf, that may be plainly visible under LM inspection can be quantified measuring the THz absorption, mainly proportional to the water content, as previously demonstrated.^45^

### 3.2 Cellular effects of THz radiation and potential biotechnological applications

Overall, the results of the use of THz radiation seem to generate controversy regarding the effect of FIR radiation on biological processes such as molecular polymerisation and demethylation, and its capability to generate hyperthermia (Table 1). In fact, it has been reported that the exposure for 240 min to CW radiation at *ν* = 2.52 THz, with an irradiance of 636 mW/cm^2^, induced the expression of cell growth factors in Jurkat cells,^46^ while in another study, a shorter exposure of 20–40 min with a reduced irradiance of 227 mW/cm^2^ caused, on the same cells, death by apoptosis and necrosis.^46^ Obviously, the comparison between different experimental setups might contribute to explain these inconsistencies.

Further experiments on human HEK001 cells exposed to THz waves (*ν* = 1.4 THz, *ν* = 2.52 THz and *ν* = 3.11 THz) have highlighted the potential to increase the expression of different genes at different frequencies, while reducing the expression of others, with mechanisms different than thermalisation since mRNAs analysis revealed that genes related to heat shock were not altered.^47^

Many studies on human cell lines seem to suggest that if the THz irradiance is kept below 1 mW/cm^2^, or limited to a few mW/cm^2^, the exposure to both pulsed and CW THz frequencies has no effect on cell types as diverse as lymphocytes, fibroblasts (NB1RGB), keratinocytes (NHK) and corneal epithelial cells (HCE‐T).^46^

Additionally, it has been shown that THz radiation can act on hydrogen bonds that stabilise the DNA structure, influencing the dynamics of ‘DNA breathing’ with the creation of a permanent opening in localised base pairs.^48^ It has been suggested that this coupling between electromagnetic field and DNA at the site of a promoter sequence can influence the transcription or repression of genes and ultimately influence cell differentiation into specific phenotypes.^49^ The development of accurate protocols to control expression of specific DNA sequences could pave the way for the adoption of THz genomic control as a complement to other well developed biotechnological techniques such as CRISPR‐Cas9.

In conclusion, to minimise collateral effects of THz radiation, similarly to the case of LM, NIR or other modalities, attention has to be paid to the total delivered dose, the mode (CW or pulsed) and the peak irradiance (W/cm^2^).

### 3.3 Considerations for the development of faster and higher‐resolution methods for THz imaging

One of the bottlenecks for the implementation of THz medical imaging systems is the acquisition speed. In the examined systems, the image acquisition of an area having a few squared centimetre size, with spatial resolution of 250 μm, required up to 200 s, even with a line scanner,^41^ or up to 10 min with a pixel size of 500 μm.^50^ Unlike laser‐scanning LM applications such as confocal or multiphoton, THz still lacks widely available optics and fast technologies to scan a sample, although alternative methods to acquire images that might speed up the acquisition with point‐like detectors may be applied. For instance, it has been shown that the use of single‐pixel detector and spatial modulation of THz, obtained photoexciting a silicon wafer with a patterned UV laser, allows the acquisition of small raster images of 32 × 32 pixels, with an acquisition speed of 6 frames per second.^51^

On the other hand, the problem of the scarce resolving power of wavelengths corresponding to THz radiation has been tackled by several studies. Some approaches are based on the reduction of the illumination spot size. For instance, it has been shown that an enhancement of the image contrast of more than 4 times can be achieved by positioning a solid dielectric cube in front of the sample; the focussed beam, called ‘terajet’, permits an improvement in resolution due to higher field intensity in a diffraction spot having a reduced full width at half maximum (FWHM) of 0.55 λ , instead of the 0.67 λ that corresponds to a dry immersion medium.^20^ Another approach used a solid immersion silicon lens between the objective and its image plane. The coupling of the aspherical singlet objective and the truncated hemisphere generates an evanescent field with 0.35 λ spot size, and a resolution enhancement of 2.43 times.^52^

An additional approach to improve the resolving power of THz systems is near‐field excitation, which requires the application of a hollow tip with a small aperture in the focus position of the beam; in one of the first adoptions, it reduced spot size allowing the resolution of 50 μm or λ /4 of the used radiation.^53^ In further developments, near‐field interactions between the sample and the tip of atomic force microscopes, or PCA microprobes, have been exploited to achieve micron and nanometre resolution in imaging and spectroscopic applications. In THz scattering‐type scanning near‐field optical microscopy (THz s‐SNOM),^54^ the electric permittivity of the sample surface can be measured with a resolution of tens of nanometres, measuring the light scattered by the tip. Conversely, the current induced between the sample surface and the probe by a strong THz field allows measurement of the local carrier density with sub‐nanometre resolution in THz scanning tunnelling microscopy (THz‐STM).^55^ THz s‐SNOM and THz‐STM have been applied so far to study metals, semiconductors or biological molecules on surfaces.

A recent combination of near‐field and THz‐TDS allowed to achieve resolution below 100 nm in the sub‐surface analysis of metallic‐dielectric slab,^56^ or, only with reconstructive imaging, a resolution of the order of several nanometres.^57^ This suggests that the adoption of highly precise positioning devices can benefit the improvement in resolution, especially if, as shown by other studies in the time domain, the Nyquist sampling of the THz signal is coupled to other steps in the signal processing such as padding and denoising.^58^

## 4 CONCLUSIONS

How the terahertz frequency range can be used in microscopy to achieve subcellular resolution and allow imaging of biological samples is determined by a combination of factors, including the availability of compact optics and system design,^40^ sufficiently powerful sources emitting at the desired wavelength,^39^ some knowledge of the sample to be imaged, physical models/approximations that can simplify the analysis (such as the effective‐medium theory^32^, ^45^), and an appropriate choice of possible correlative methods – of which the most straightforward is light microscopy.^41^, ^50^

Furthermore, lens design improvements,^52^ the use of accurate positioning hardware and customised data/image processing pipeline^57^ can help to tackle the issue deriving from the Abbe diffraction limit at long wavelengths.

Deconvolution methods applied to THz imaging^58^ can hugely contribute to improving the resolving power of these long wavelengths that offer advantages in terms of functional imaging (molecular fingerprint, relaxation dynamics etc.) of biological samples and non‐ionising radiation for clinical use. However, more work is required to push the boundary of THz resolution towards subcellular level and render THz microscopy suitable as a correlative technique for life sciences.

In this review, we have described the most commonly adopted imaging modalities for biological and clinical samples. More in‐depth attention has been given to the use of THz imaging for biomedical applications, since the physics of long wavelengths is well understood, but the promising biological applications have not been yet fully explored. In our view, the microscopy field can hugely benefit by the implementation of THz in routine acquisition platforms such as wide‐field and laser‐scanning systems.
